# Enamel surface alterations after repeated conditioning with HCl

**DOI:** 10.1186/s13005-015-0089-2

**Published:** 2015-09-25

**Authors:** W H Arnold, B. Haddad, K. Schaper, K. Hagemann, C. Lippold, Gh. Danesh

**Affiliations:** Department of Biological and Material Sciences in Dentistry, Faculty of Health, School of Dentistry, Witten/Herdecke University, Witten, Germany; Department of Orthodontics, Faculty of Health, School of Dentistry, Witten/Herdecke University, Witten, Germany; Department of Medical Biometry and Epidemiology, Faculty of Health, School of Medicine, Witten/Herdecke University, Witten, Germany; Private Praxis for Orthodontics, Essen, Germany; Department of Orthodontics, University Medical Centre of Muenster, Muenster, Germany

## Abstract

**Background:**

The aim of this study was to investigate the influence of etching time with 15 % hydrochloric acid (HCl) on the enamel surface destruction by studying the resulting roughness and erosion depth.

**Methods:**

The vestibular surfaces of 12 extracted, caries free human incisors were divided into four quadrants, and each quadrant was etched with 15 % HCl for different numbers of etching cycles (1×2, 2×2, 3×2 and 4×2 min). Surface roughness and erosion depth were measured quantitatively with optical profilometry, and the surface morphology was imaged with scanning electron microscopy (SEM).

**Results:**

After two minutes of 15 % HCl application a median enamel substance loss of 34.02 μm was observed. Lengthening of etching time (2×2, 3×2 and 4×2 min) resulted in significantly increase in erosion depth to each additionally, between 13.28 -15.16 μm (*p* < 0.05) ending up in a total median enamel surface loss of 77 μm. Regarding surface roughness no significant (*p* > 0.05) difference was found between unetched enamel and the etched enamel surfaces.

**Conclusion:**

Repeated 15 % HCl conditioning of the enamel surface increases the depth of the etched surface erosion. However, the total erosion depth is rather shallow and therefore negligible.

## Background

A common problem in orthodontic treatment with fixed appliance is the development of white spot lesions [1]. Orthodontic treatment with brackets creates retention sites for plaque, which subsequently promotes the development of white spot lesions [2]. Subsurface demineralization increases the pore volume of enamel, which changes the optical refraction of enamel and results in the white, opaque color of white spot lesions [3, 4]. Various white spot lesion treatment strategies exist. The first is preventive oral hygiene education and motivation of the patient, followed by fluoridation of the enamel surface [4]. Invasive treatment is the last option in the treatment of white spot lesions. During the past decade, a micro-invasive infiltration technique for white spot lesions using Icon® (DMG, Hamburg, Germany) has been developed, which uses a complete resin infiltration of initial carious lesions and hinders further caries progression [5]. Application of this micro-invasive infiltration technique is recommended for non-cavitated lesions up to ICIDAS III [6]. It masks white spot lesions and closes lesion pores, thus arresting further demineralization [7, 8].

A major challenge for resin infiltration is the need to remove the hypermineralized surface layer of the lesion during enamel etching [9–11]. Surface conditioning is necessary because this hypermineralized surface layer hampers resin infiltration into the lesion [9, 10, 12]. Comparative studies showed that for surface conditioning of the enamel, 15 % hydrochloric acid (HCl) has to be used. Other concentrations (5 % HCl) or acids (37 % phosphoric acid) are not effective enough [9, 13].

According to the manufacturer’s instructions, a 2 min etching time is recommended. If the white spot is still visible, the etching should be repeated no more than 2 times. However, repeated etching of the enamel surface results in further destruction of the enamel prisms. Further studies of repeated HCl application on the enamel surface are necessary to determine the limitations of acid etching with 15 % HCl.

The aim of this investigation was to study the surface morphology of etched enamel with scanning electron microscopy (SEM) and quantitative profilometry after repeated applications of 15 % HCl.

## Methods

### Tooth preparation

Twelve extracted, caries free human incisors were stored in 0.9 % NaCl solution containing 0.1 % thymol. Before storage all teeth were cleaned with a toothbrush under running tape water. Tooth collection was approved by the ethical committee (116/2013), and verbal consent was obtained from the patients. Teeth used in the present study were extracted due to periodontal reasons. An ICDAS score of 0 on the buccal enamel surface was determined visually.

The teeth were covered with acid resistant nail varnish (BIOCURA® BEAUTY, Nailvarnish, Maxim Markenprodukte GmbH & Co. KG, Pulheim-Brauweiler, Germany) leaving four 2×2 mm windows on the vestibular surface. Each window was treated with 15 % hydrochloric acid (HCl, Icon-Etch®, DMG, Hamburg, Germany) for different periods of time. The time periods were 2, 2×2, 3×2 and 4×2 min. After each etching cycle, the enamel surface was rinsed with tap water for 30 s and air dried before the next application of HCl. All teeth were then stored in NaCl/thymol solution until further examination.

### Scanning electron microscopy (SEM)

The roots of the teeth were removed using a diamond saw, and the nail varnish was removed with acetone. All teeth were then dried in graded acetone, mounted on specimen holders, and sputtered with gold palladium (Bal-TEC SCD 050 Sputter Coater, Balzer, Lichtenstein). The etched windows were imaged with a scanning electron microscope (Zeiss Sigma VP, Carl Zeiss, Oberkochen, Germany) using a 20 kV acceleration voltage and an SE detector.

### Profilometry

Surface roughness measurements were performed with an optical profilometer (InfiniteFocus^©^,G3, Alicona^©^ Imaging GmbH, Graz, Austria) using a vertical resolution of 450 nm and an Lc of 25. The depth of each etched window was measured using the single step height measurement function of the software. In each window, 20 roughness measurements were taken to determine the area scale fractal complexity (asfc), which is related to the fractal dimension of the surface texture [14], and 20 depth measurements were taken in the window.

### Statistical analysis

Prior to the experiments a power analysis was carried out with an alpha of 0.05 and a power of 0.8 (sigma 1 = 10.00, sigma 2 = 11.35; mean 1 = 35.95, mean 2 = 49.37) which resulted in a sample size of 10. Power analysis was calculated using Axum 7 (Mathsoft, Cambridge, Massachusetts, USA). The statistical calculations were performed with the SPSS (IBM Corporation, Amronk, NY, USA; Rel. 21) statistical software. The non-parametric Wilcoxon sign test for related variables was used with a *p* < 0.05 to determine significance. Comparison of the differences between the time intervals was performed with the Friedman-test.

## Results

Power analysis resulted in a minimum sample size of 10 teeth. Therefore, 14 teeth were used for this investigation.

**Fig. 1:**
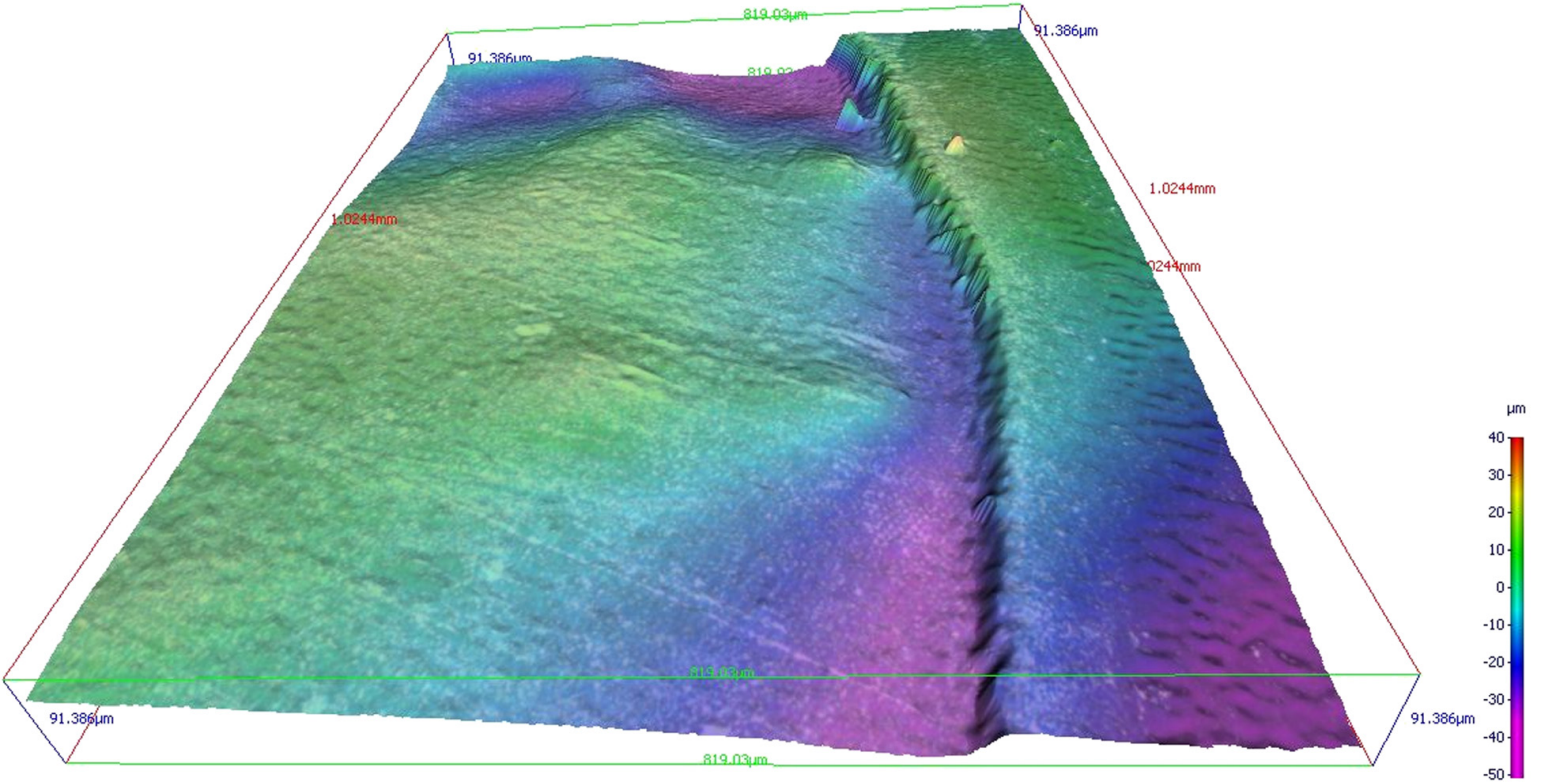
3D image of the amount of enamel surface dissolution after etching for 2×2 min with 15 % HCl

**Fig. 2:**
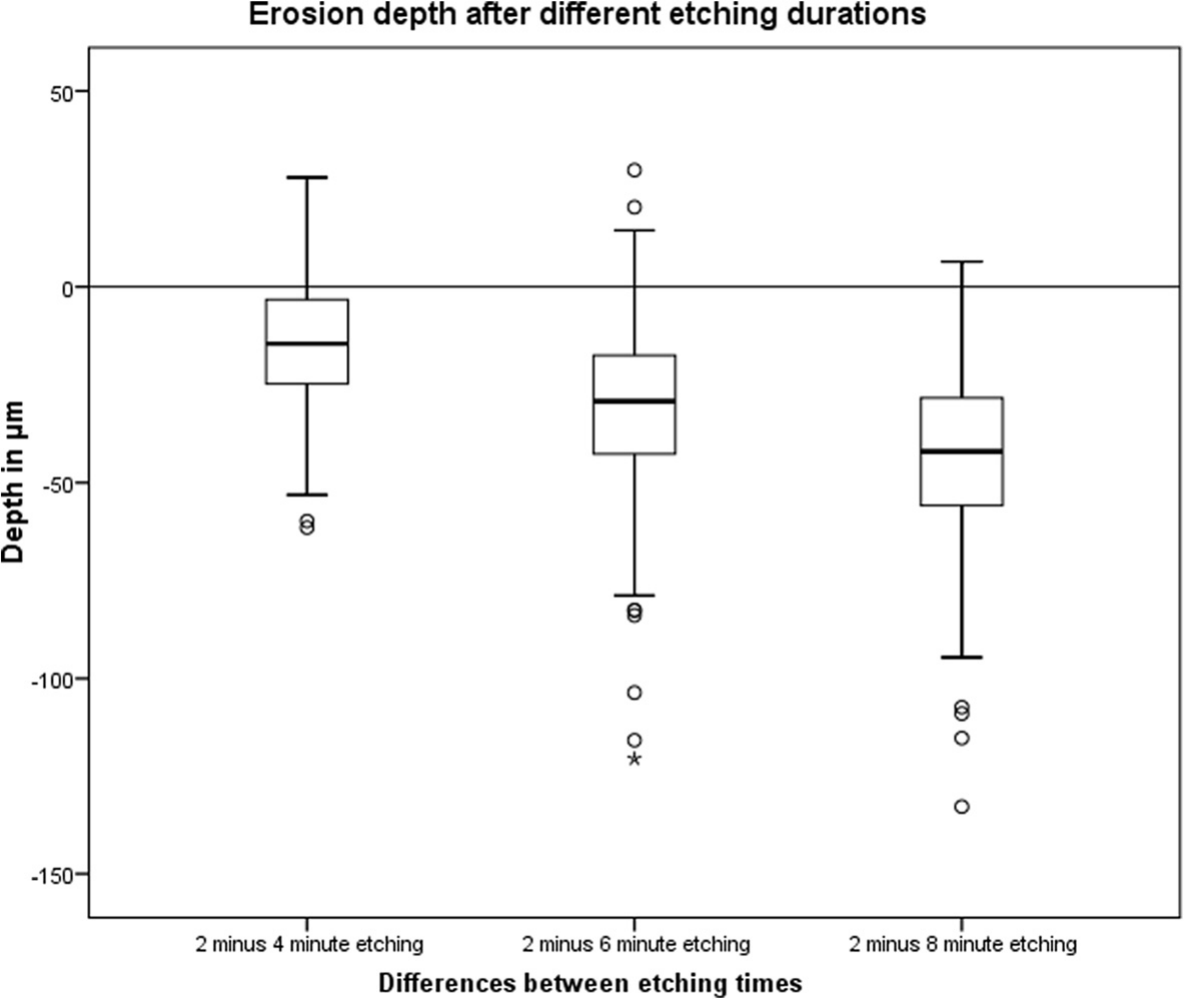
Comparison of the gradient of the enamel lesion depth after different etching duration. The differences are calculated as follows: The depth of the 2 min etched window was subtracted from the depth of the 2×2, 2×4 and 2×6 min etches windows

Profilometry demonstrated shallow, etched surfaces with steep ledges (Fig. 1). Table 1 displays the depth of the etched enamel surfaces after different etching durations. The depth of the etched superficial defects increased significantly (*p* < 0.05) with the etching duration. The largest substance loss was found after two minutes, with a median loss of enamel substance of 34.02 μm (Table 1). Lengthening the etching time (2×2, 3×2 and 4×2 min) resulted in a significant increase (*p* < 0.05) in erosion depth (13, 28–15, 34 μm) and is shown in Fig. 2.

**Table 1:** Influence of etching time on the depth of the enamel erosion depth [μm]

Conditioning time	2 min	2×2 min	2×3 min	2×4 min
Mean	35.95	49.37	66.46	79.22
Standard devation	10.00	11.35	18.06	19.79
Median	34.11*	49.36*	64.28*	77.56*
Minimum	17.75	22.52	32.79	39.91
Maximum	71.34	92.47	151.51	150.58
25 % percentile	28.40	41.77	54.94	66.09
75 % percentile	41.10	55.42	73.67	90.72

Values with the superscript asterisk were significantly different (*p* < 0.05)

The roughness data are summarized in Table 2. While comparing the profilometric measurements for roughness, no significant difference (*p* < 0.05) was found between the untreated control surfaces and the etched surfaces (Table 2).

**Table 2:** Influence of the etching time on the roughness of the enamel surface

Conditioning time	controls	2 min	2×2 min	2×3 min	2×4 min
Mean	3.26	3.49	2.83	2.99	3.64
Standard deviation	0.72	1.41	1.87	1.72	1.42
Median	3.14	3.22	2.91	3.88	3.46
Minimum	2.03	1.37	0.41	1.37	2.18
Maximum	4.49	6.39	6.86	6.81	6.88
25 % percentile	2.67	2.70	1.10	2.00	2.54
75 % percentile	3.68	4.65	4.21	5.31	4.42

All numbers are asfc values

**Fig. 3:**
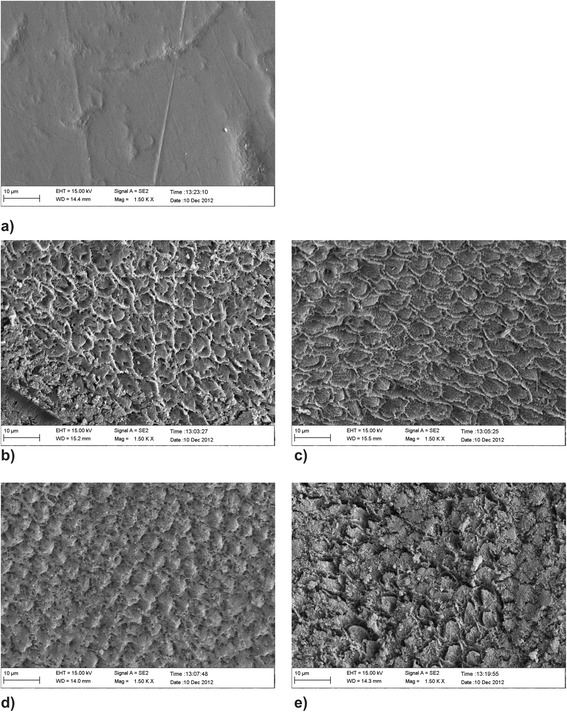
(**a-e**) SEM photo of unetched enamel surface showing a relative smooth surface whith prism cores. **b** SEM photo of the enamel surface pattern after etching for 2 min with 15 % HCl showing dissolution of the prism core and an intact prism periphery; **c**) surface pattern after etching for 2×2 min with 15 % HCl showing little dissolution of the prism core and the periphery; **d**) surface pattern after etching for 3*2 min with 15 % HCl showing dissolution of the prism periphery; **e**) surface pattern after etching for 4*2 min with 15 % HCl showing destruction of the enamel prisms core and periphery

SEM revealed four different etching patterns, which were found in all windows independent of the etching time. Pattern one was homogeneous with dissolution of the prism core and relatively intact prism peripheries (Fig. 3a). Pattern two showed more variation in the amount of dissolution of the prism core and minor dissolution of the prism periphery (Fig. 3b). Pattern three was characterized by a deep prism periphery (Fig. 3c), and pattern four showed variable dissolution of the prism core and prism periphery (Fig. 3d).

**Fig. 4:**
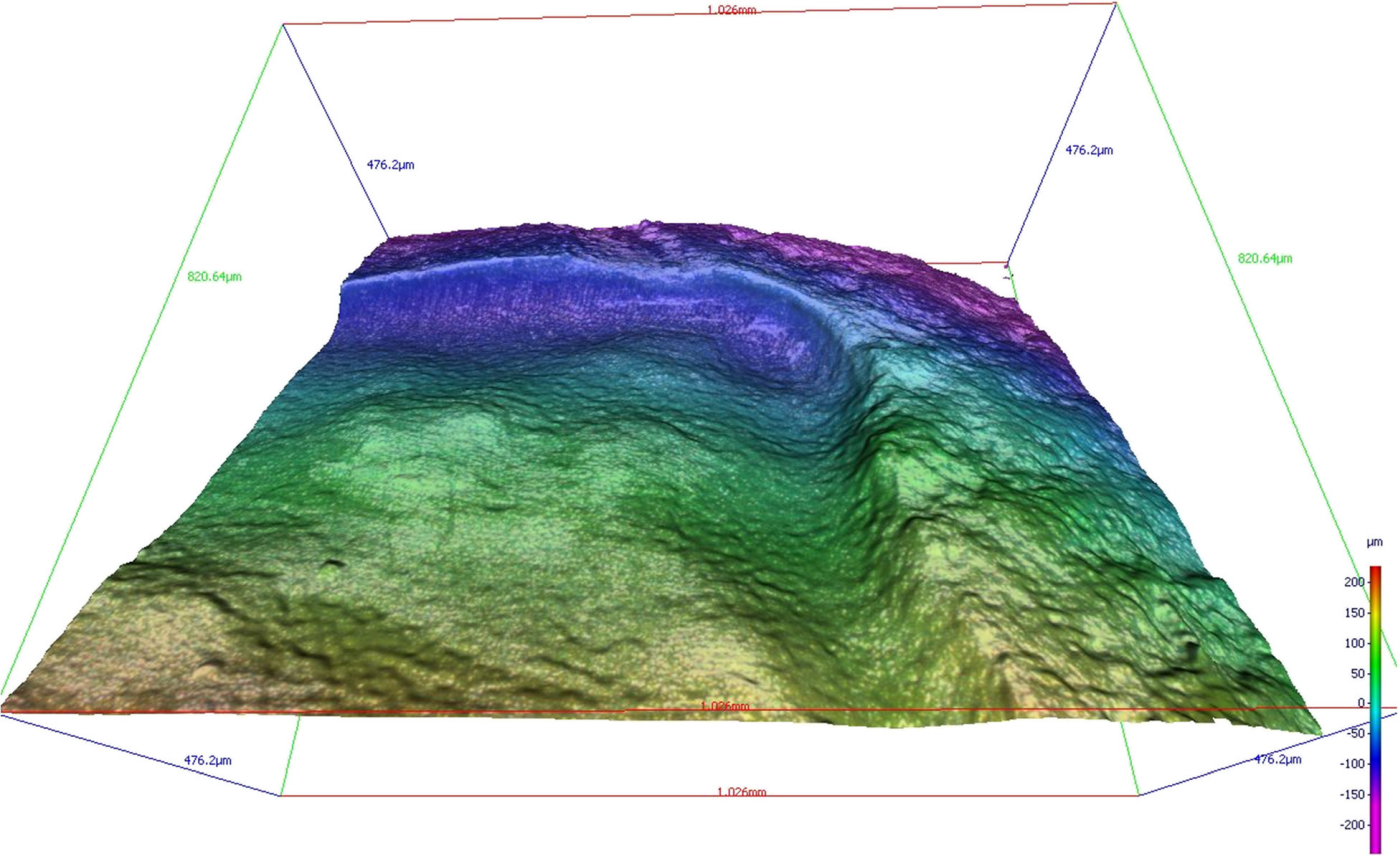
3D-reconstruction using Alicona Infinite Focus^©^. The etched enamel surface is deeper in the periphery than the central area

Moreover, the samples examined with profilometry showed a greater depth in the periphery than in the central portion of the conditioned area (Fig. 4).

## Discussion

By definition, initial white spot carious lesions (WSL) that are stage ICDAS I and ICDAS II are characterized by a hypermineralized surface layer and a demineralized subsurface body [15]. This hypermineralized surface layer hampers resin infiltration into the underlying subsurface demineralization of the lesion and has to be removed before resin infiltration [9–12]. Phosphoric acid does not adequately remove the surface layer, but it was demonstrated that 15 % HCl for 90–120 s sufficiently removes the hypermineralized surface layer [9] and enables resin infiltration into the body of the lesion. Fifteen percent HCl is used as a pre-treatment in the micro-invasive therapy of WSL by Icon ^©^ (DMG, Hamburg, Germany) as an alternative option for non-cavitated lesions [9, 11, 12, 16]. HCl is rarely used in dentistry because of its strong acidity [17]. HCl quickly destroys the crystalline structure of hydroxyapatite, and repetitive etching enhances this effect. This observation is important for the areas of sound enamel adjacent to the lesion. Therefore, one aim of this in vitro study was to evaluate the erosion depth of etched enamel after repeated 15 % HCl applications.

Teeth used in the present study were extracted for periodontal reasons. Furthermore, an ICDAS score of 0 on the buccal enamel surface was determined visually. It is possible that areas with an ICDAS score of 1 were present in the 12 tooth samples. Regardless, it is unlikely that the results were distorted by this because those types of lesions do not exceed a depth of 40 microns [9]. An erosion depth of 34 μm was observed in 75 % of tooth samples after conditioning with HCl for 2 min.

In the present study, optical profilometry was used. In contrast to tactile profilometry, this method allows for a zero-contact measurement of the samples in a non-destructive way [18]. The profilometric experiments of this study demonstrated that repeated application of 15 % HCl removes the surface layer with a median depth of 77.56 μm. In this study, a median enamel substance loss of 34.02 μm occurred after treatment for two minutes with 15 % HCl. This finding is similar to the findings of Meyer-Lueckel et al. [9]. Lengthening the etching time (2×2, 3×2 and 4×2 min) resulted in a significant increase in the depth of the lesion (13, 28–15, 34 μm).

To clinically improve esthetic results, studies have recommended etching times of more than 120 s [19]. White spot lesions that have been present for a longer period of time and deeper lesions with a thicker intact surface layer require more conditioning time [19]. This raises the question of whether the amount of enamel loss over multiple cycles of conditioning is clinically significant. The average thickness of the enamel of the upper central and lateral incisors ranges from approximately 0.5 mm in the cervical third to more than 1 mm in the incisal third [20, 21]. Bailey and Christen found that a 30 % reduction of the enamel layer is clinically acceptable [22]. For 960 measurements in the present study, only 11 had a reduction of more than 120 μm. Seven of those were in the 8 min group. After 4×2 min of etching time with HCl, an etching depth of approximately 77 μm was achieved in this study, which is well below the threshold of clinical significance.

In general, when acid-etching a white spot lesion with phosphoric acid or hydrochloric acid, it is difficult to avoid etching the adjacent sound enamel. An in vitro study showed that samples treated with infiltrating resin were protected against acid dissolution (even in sound enamel, which is not a typical substrate for the infiltrate) [23].

Some clinical studies reported good results for masking WSL with infiltrates [7, 19, 24, 25]. In this context, Knoesel et al. [19] reported adequate esthetic outcomes regarding the durability of infiltration after 6 months in vivo. Compared to the microabrasion technique, which can remove up to 360 μm of enamel during the treatment of WSL [17], the pre-treatment of the surface with 15 % HCl prior to infiltration is gentler.

Another aim of this study was to evaluate the surface roughness after repeated HCl applications. In contrast to surface destruction, etching time did not influence the surface roughness. A significant difference in the surface roughness was found between the 2 min and 4×2 min groups, which decreases the asfc units and reflects a smoother surface. In the other groups, no significant difference in roughness was observed.

This finding is in contrast to a previous study [26]. Takeya reported an increase in surface roughness with increased etching times and that the roughness of the enamel surface significantly increased after increasing the conditioning periods from 15 to 30 and 60 to 120 s. The acids used in the study of Takeya, however, were not hydrochloric acid but phosphoric, citric and pyruvic acid [26]. Furthermore, the reason for the contradictory results may be the different method of measuring roughness. Takeya used a tactile roughness measurement method, which is somewhat destructive. Thus, those measurements may be influenced by the mechanical resistance of the etched prism surfaces.

Other studies showed that after microabrasion with 18 % hydrochloric acid (compared to 37 % phosphoric acid), the roughness of the enamel decreased, and the amount of demineralization simultaneously increased [27]. Conversely, in vitro investigations showed that surfaces treated with the caries infiltrate appeared demineralized and showed higher surface roughness compared to surfaces treated with conventional adhesives or a combination of both [23, 28]. In practice, this should be therapeutically taken into account.

The SEM images showed different etching patterns, which resulted from the variable dissolution of the prism cores and prism peripheries. These differences in etching patterns are not due to the duration of etching with 15 % HCl, but rather the difference in enamel prism morphology.

Another finding in this study was that the profilometrically analyzed samples showed a greater depth in the periphery of the etched area than in the central area. It was assumed that the hydrochloric acid was locked into the applied area for soak times of 2 min or more, but due to the convex surface of the tooth, the acid flowed into the periphery of the square delineated by the nail varnish. The size of the lesion may limit the ability to condition the surface, increasing the clinical difficulty of treatment. When lesions are located near adjacent teeth, preparatory precautions are mandatory to protect nearby tooth structures.

### Conclusions

In this study, we showed that the depth of the etched surface layer is dependent upon the etching time. Repeated pretreatment with 15 % HCl resulted in a reduction of surface roughness. Pre-treating with 15 % HCl prior to infiltration results in shallow enamel surface erosions. The long-term effects 15 % HCl etching prior to the infiltration treatment should be followed up with continuous clinical and scientific research.

## Abbreviations

ICDAS: International Caries Detection and Assessment System
SEM: Scanning electron microscope
Ra: Average roughness of profile
asfc: Area-scale-fractal-complexity
SPSS: Statistical package for social sciences
